# Clinical Features and Prognostic Impact of Coexpression Modules Constructed by WGCNA for Diffuse Large B-Cell Lymphoma

**DOI:** 10.1155/2020/7947208

**Published:** 2020-06-07

**Authors:** Jianjun Xiao, Xuemei Wang, Haitao Bai

**Affiliations:** ^1^Department of Oncology, Shaoxing Second Hospital, No. 123 Yan'an Road, Yuecheng District, Shaoxing, 312000 Zhejiang Province, China; ^2^Department of Cardiology, Obstetrics and Gynecology Hospital of Fudan University, No. 419, Fangxie Road, Huangpu District, Shanghai 200011, China; ^3^Department of Haematology, Shanghai General Hospital of Nanjing Medical University, No. 100 Haining Road, Hongkou District, Shanghai 200080, China

## Abstract

**Objective:**

Diffuse large B-cell lymphoma (DLBCL) is a highly aggressive malignant tumor, accounting for 30-40% of non-Hodgkin's lymphoma. Our aim was to construct novel prognostic models of candidate genes based on clinical features.

**Methods:**

RNA-seq and clinical data of DLBCL were retrieved from TCGA database. Coexpression modules were constructed by WGCNA. Then, we investigated the interactions between modules and clinical features. By overall survival analysis, prognostic candidate genes from modules of interest were identified. A coexpression network of prognostic candidate genes was then constructed through WGCNA. GEPIA was used to analyze the expression of a candidate gene between DLBCL and normal samples.

**Results:**

19 coexpression modules were constructed by 12813 genes from 52 DLBCL samples. The number of genes in modules ranged from 34 to 5457. We found that the purple module was significantly related with histological type (*p* value = 1*e*-04). Overall survival analysis revealed that MAFA-AS1, hsa-mir-338, and hsa-mir-891a were related with prognosis of DLBCL (*p* value = 0.027, 0.039, and 0.022, respectively). A coexpression network was constructed for the three prognostic genes. MAFA-AS1 was interacted with 36 genes, hsa-mir-891a was interacted with 11 genes, while no gene showed interaction with hsa-mir-338. Using GEPIA, we found that MAFA-AS1 showed low expression in DLBCL samples (*p* < 0.01).

**Conclusion:**

We constructed a coexpression module related with histological type and identified three candidate genes (MAFA-AS1, hsa-mir-338, and hsa-mir-891a) that possessed potential value as prognostic biomarkers and therapeutic targets of DLBCL.

## 1. Introduction

DLBCL is a highly aggressive malignant tumor originating from mature B-cells, accounting for 30-40% of non-Hodgkin's lymphoma [1, 2]. Patients with DLBCL usually have a poor prognosis due to ineffective primary and second-line therapy or recurrence after stem cell transplantation [3]. Therefore, easily applicable prognostic parameters are necessary for clinicians, especially since new molecular markers have not yet entered clinical routines [4]. The International Prognostic Index (IPI) is the most common tool for risk stratification in DLBCL. However, due to improved treatment options, pathobiology, and life expectancy of patients with DLBCL, IPI has been challenged [5]. Therefore, it is necessary to propose novel prognostic models based on clinical features and prognostic biomarkers.

As a molecular heterogeneous disease composed of different histopathologic and genetic subtypes, genetics of DLBCL has clinical implications for patient risk prediction and treatment [6]. Molecular traits are increasingly being used to guide DLBCL drug development, predict patients' clinical outcomes, and make treatment plans [7]. Therefore, the collection and evaluation of molecular features in clinical samples are critical to improve the prognosis of patients with DLBCL. Genomic studies have revealed a large number of mutant genes in DLBCL; their clinical significance remains unclear.

As a powerful method for transcriptomics analysis, RNA sequencing (RNA-seq) has been widely used to explore gene function and biological patterns, as well as to find candidate drug targets and to identify biomarkers for predicting disease risk and prognosis [8, 9]. The Cancer Genome Atlas (TCGA) has produced RNA-seq data, which provides an unprecedented opportunity for cancer biology. Weighted gene coexpression network analysis (WGCNA) is a systematic biological method, which is widely used to generate gene coexpression networks [10]. Instead of linking thousands of genes to physiological characteristics, it focuses more on the relationship between several modules and features [11]. It provides a specific measure for clinical prediction of DLBCL diagnosis and developing new treatment strategies [12]. Therefore, WGCNA can explore hidden biological patterns. The method has been used to analyze many kinds of diseases, such as breast cancer, uveal melanoma, gastric cancer, and colon cancer [13–16]. However, there is no study on clinical modules of DLBCL using the WGCNA method.

In the present study, coexpression modules were constructed by WGCNA. After investigating the correlations between modules and clinical features, a module of interest was identified for functional enrichment analysis. We found three candidate genes (MAFA-AS1, hsa-mir-891a, and hsa-mir-338) that were related with prognosis of DLBCL by overall survival analysis. And MAFA-AS1 had low expression in DLBCL by GEPIA.

## 2. Materials and Methods

### 2.1. Data Processing

RNA-seq and clinical data of lymphoid neoplasm diffuse large B-cell lymphoma patients were downloaded from TCGA data repository (https://cancergenome.nih.gov/). The gene expression level was normalized as fragments per kilobase of transcript per million mapped reads (FPKM) using Robust Multiarray Average (RMA) algorithm. The miRNA expression level was measured as RPM. Clinical information included clinical TNM stage, histologic grade, age, gender, and survival information. As genes with little variation in expression usually represent noise, the most variant genes were filtered. Gene variables were measured by median absolute deviation (MAD).

### 2.2. Construction of Gene Coexpression Network

Gene coexpression network was constructed using WGCNA package in R [17]. Power values were screened out by the WGCNA algorithm. Firstly, gene expression similarity matrix S = (*s*_*ij*_) was constructed via calculating the absolute value of Pearson correlation coefficient between two genes. The formula was listed as below:

(1) $$\begin{array}{c} s_{ij}=\left|\frac{1+\text{cor}\left({\text{x}}_{\text{i}},{\text{x}}_{\text{j}}\right)}{2}\right|, \end{array}$$

where x_i_ and x_j_ were vectors of expression value for gene *i* and *j* and *s*_*ij*_ represented the Pearson correlation coefficient of gene *i* and gene *j*.

Next, gene expression similarity matrix was transformed into adjacency matrix a_ij_. The formula was a_ij_ = |*s*_*ij*_|^*β*^. To further identify functional modules in the coexpression network, the adjacent matrix was transformed into a topological overlap matrix (TOM), and the corresponding dissimilarity (1-TOM) was calculated.

(2) $$\begin{array}{c} {\text{tom}}_{ij}=\frac{\sum_{\mu \neq i,j}a_{i\mu }a_{\mu j}+a_{ij}}{\min\left(\sum_{\mu }a_{i\mu },\sum_{\mu }a_{\mu j}\right)+1-a_{ij}}, \end{array}$$

where *α* was the weighted adjacency matrix given by a_ij_ = |*s*_*ij*_|^*β*^ and *β* =5 was the soft threshold power.

(3) $$\begin{array}{c} D=\left(\begin{array}{c} d_{1}\\ d_{2}\\ \vdots \\ d_{n} \end{array}\right), \end{array}$$

where *D* expressed the degree of dissimilarity gene expression in different samples.

Scale independence and average connectivity analysis of modules with different power values was performed by gradient test (power value ranging from 1 to 20). Appropriate power value was determined when the scale independence value was equal to 0.9. The WGCNA algorithm was then used to construct the coexpression network and extract the gene information in the most relevant module. The candidate network was selected according to the coexpression weight > 2.5.

### 2.3. Correlations between MEs and External Clinical Data

The correlations between modules and clinical features were analyzed using WGCNA. Module eigengene (ME) can summarize the gene expression profiles, and the formula was as follows:

(4) $$\begin{array}{c} {\text{ME}}_{i}^{q}=\text{princomp}\left(x_{ij}^{\left(q\right)}\right), \end{array}$$

where *q* represents the *q*th module.

We calculated the correlations between MEs and external clinical data as the module membership (MM). *p* < 0.05 was statistically significant. The genes in the most relevant module were chosen as candidate genes, as follows:

(5) $$\begin{array}{c} {\text{MM}}_{i}^{q}=\text{cor}\left(x_{i},{\text{ME}}_{i}^{q}\right), \end{array}$$

where ME_*i*_^*q*^ means the identification of the *i*th gene in the *q*th module.

We calculated the correlations between MEs and external clinical data. A *p* value < 0.05 was statistically significant. The genes in the most relevant module were chosen as candidate genes.

### 2.4. Functional Enrichment Analysis

To explore the potential biological themes and pathways of genes from modules of interest, the clusterProfiler package in R was used to annotate and visualize Gene Ontology (GO) terms (including biological processes, molecular functions, and cellular components) and Kyoto Encyclopedia of Genes and Genomes (KEGG) pathways [18]. A *p* value < 0.05 was considered significant pathways.

### 2.5. Prognostic Analysis of Candidate Module Genes

Then, we further assessed the prognostic value of candidate genes by overall survival analysis. Log-rank tests were used to select prognosis-related genes from candidate genes. Survival package was used to carry out log-rank tests and survminer package was used to plot Kaplan–Meier survival curves.

### 2.6. Candidate Gene Coexpression Network Construction

WGCNA can evaluate coexpression information of genes. Through survival analysis, we obtained the core genes related with prognosis. Moreover, we constructed a coexpression network of prognosis-related genes through WGCNA.

### 2.7. Candidate Gene Risk Assessment

The online database Gene Expression Profiling Interactive Analysis (GEPIA) (http://gepia.cancer-pku.cn/index.html) was used to analyze the gene expression between cancer and normal samples [19]. As an interactive web, GEPIA can analyze the RNA-seq expression including TCGA datasets and Genotype-Tissue Expression (GTEx) datasets [20]. Using GEPIA, we analyzed candidate genes related with prognosis between cancer and normal samples from TCGA.

### 2.8. Reverse-Transcription Quantitative PCR (RT-qPCR)

Total RNA was extracted from plasma of 11 patients with DLBCL and 11 healthy participates using Trizol. To examine hsa-miR-338-3p, hsa-mir-338-5p, and hsa-miR-891a-5p expression, cDNA was synthesized with the miScript Reverse Transcription Kit (Qiagen, Hilden, Germany). RT-qPCR was performed using the miScript SYBR Green PCR Kit (Qiagen). *β*-Actin served as an internal control. The relative expression levels of miRNAs were calculated with the 2^-*ΔΔ*Ct^ method. The specific primers for hsa-miR-338-3p, hsa-mir-338-5p, and hsa-miR-891a-5p were as follows: hsa-miR-338-3p, 5′-AACCGGTCCAGCATCAGTGATT-3′ (forward), 5′-GTGCAGGGTCCGAGGT-3′ (reverse); hsa-mir-338-5p, 5′-CAATATCCTGGTGCTGAGTG-3′ (forward), 5′-GTGCAGGGTCCGAGGT-3′ (reverse); and hsa-miR-891a-5p, 5′-GTGCTCGCTTCGGCAGCACATA-3′ (forward), 5′-GTGCAGGGTCCGAGGT-3′ (reverse).

## 3. Results

### 3.1. Gene Coexpression Network of DLBCL

**Figure 1:**
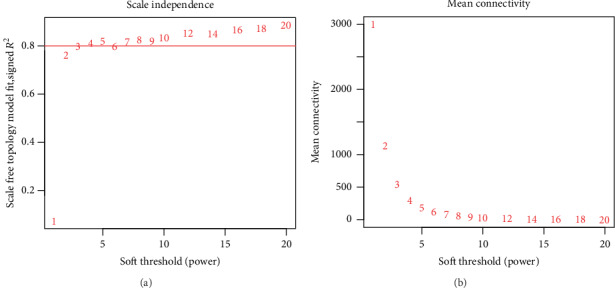
Analysis of network topology for various soft-thresholding powers: (a) the scale-free fit index as a function of the soft-thresholding power. The *x*-axis represents the soft-thresholding power and the *y*-axis represents the scale-free fit index; (b) the mean connectivity as a function of the soft-thresholding power. The *x*-axis stands for the soft-thresholding power and the *y*-axis stands for the mean connectivity.

**Figure 2:**
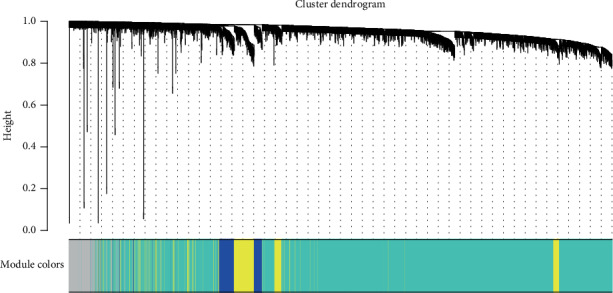
Cluster dendrogram obtained by average linkage hierarchical clustering. The color below the dendrogram demonstrates the module assignment determined by the dynamic tree cut. 19 coexpression modules were depicted in different colors. The number of genes in coexpression modules ranged from 34 to 5457.

Clinical and level-3 RNA-seq data of 51 DLBCL samples were retrieved from TCGA. The clinical features of the DLBCL samples are listed in Table 1. After module detection, 12813 most variant genes were selected for further analysis according to MAD value when the value of soft thresholding power *β* was 12, and the connectivity between genes met a scale-free network distribution (Figure 1). 19 modules were identified by hierarchical clustering and the dynamic branch cutting (Figure 2). Each module was assigned a unique color as an identifier. The number of genes in modules ranged from 34 to 5457. The grey module represented a gene set that was not assigned to any of the modules.

### 3.2. Identification of the Purple Module as the Module Most Relevant to Clinical Traits

**Figure 3:**
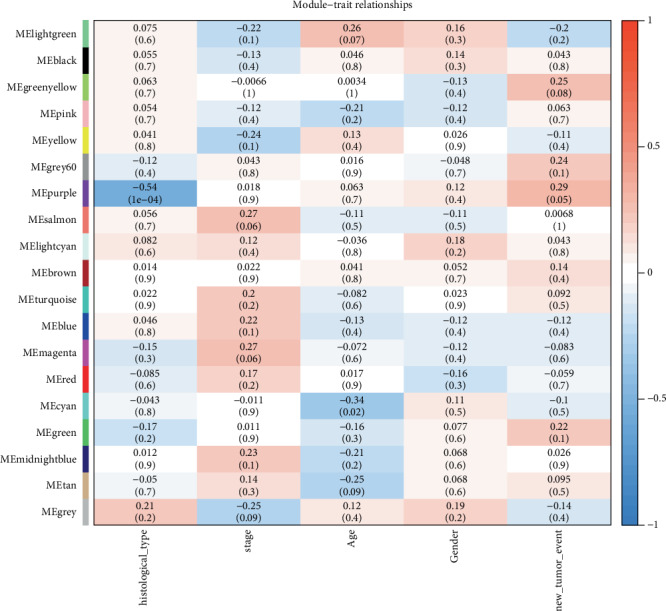
Module–trait relationships. Each row represents a module eigengene, and column represents a trait. Each cell contains the corresponding correlation and *p* value. The table is color-coded by correlation based on the color legend.

To explore the molecular mechanisms behind the trait, we identified genes associated with a certain clinical trait. In the present study, the clinical parameters of DLBCL patients, including age, clinical stage, gender, recrudescence stage, and histological type were involved in the module–trait relationship analysis. As shown in Figure 3, the purple module was closely related with histological type (*p* value = 1*e*-04) and the cyan module was associated with age (*p* value = 0.02). Therefore, the purple module was selected as module of interest for subsequent analysis.

### 3.3. Functional Enrichment Analysis of Genes in the Purple Module

**Figure 4:**
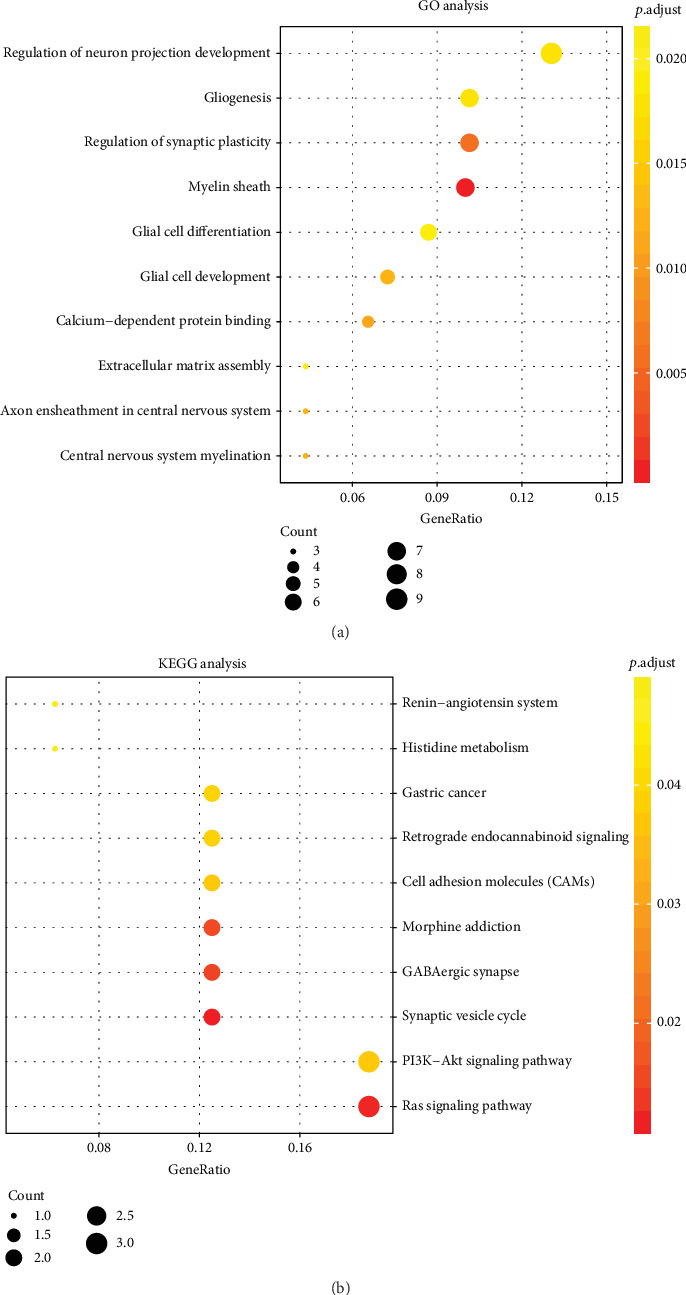
Functional enrichment analysis results of genes in the purple module: (a) the top ten GO terms of genes in the purple module; (b) KEGG pathways of genes in the purple module.

To explore potential pathways of the purple module, GO and KEGG enrichment analyses were performed on the genes from the purple module. 52 GO-enriched terms were shown Table 2. The top ten GO terms included regulation of neuron projection development, gliogenesis, regulation of synaptic plasticity, myelin sheath, glial cell differentiation, glial cell development, calcium-dependent protein binding, extracellular matrix assembly, axon ensheathment in central nervous system, and central nervous system myelination (Figure 4(a)). In the KEGG analysis, 10 pathways were enriched by genes in the purple module, including synaptic vesicle cycle, Ras signaling pathway, GABAergic synapse, morphine addiction, cell adhesion molecules (CAMs), PI3K-Akt signaling pathway, retrograde endocannabinoid signaling, gastric cancer, histidine metabolism, and renin-angiotensin system (Table 3, Figure 4(b)).

### 3.4. MAFA-AS1, hsa-mir-338, and hsa-Mir-891a as Candidate Genes Related with Prognosis of DLBCL

**Figure 5:**
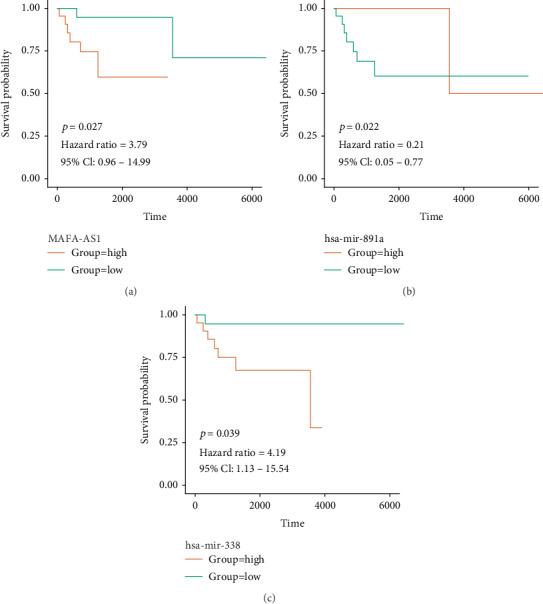
Overall survival analysis of three candidate genes: (a) MAFA-AS1; (b) hsa-mir-891a; (c) hsa-mir-338.

The purple module was closely related with histological type. Therefore, genes in the module were of great significance to evaluate the potential value. Among the 100 genes, 3 genes (MAFA-AS1, hsa-mir-338, and hsa-mir-891a) were associated with prognosis of DLBCL (Table 4). As shown in Figure 5, highly expressed MAFA-AS1 and hsa-mir-338 had shorter survival time than their low expression (*p* value = 0.027 and 0.039, respectively). And low expression of hsa-mir-891a had poorer clinical outcomes than its high expression (*p* value = 0.022).

### 3.5. Candidate Gene Coexpression Network Construction

**Figure 6:**
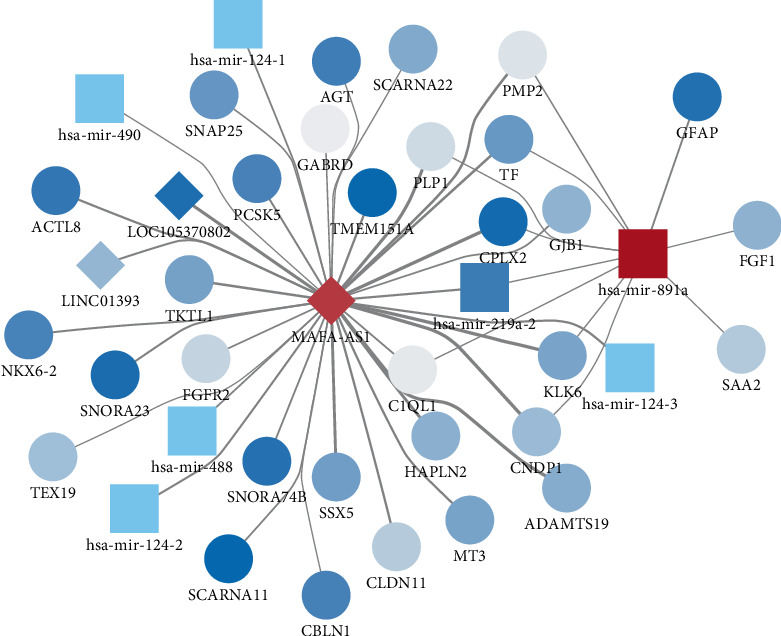
Candidate gene coexpression network construction. Round stands for mRNAs and rhombus represents miRNAs.

We constructed a coexpression network for genes with prognostic significance by WGCNA. As shown in Figure 6, MAFA-AS1 was interacted with 36 genes, hsa-mir-891a was interacted with 11 genes, while no gene showed interaction with hsa-mir-338.

### 3.6. Candidate Gene Risk Assessment

**Figure 7:**
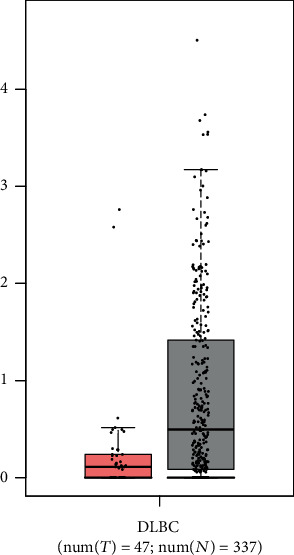
Candidate gene risk assessment using GEPIA. Red represents high expression, and gray represents low expression. There are 47 patients with DLBCL and 337 normal patients.

Using GEPIA datasets, we found that MAFA-AS1 showed low expression in DLBCL samples (*p* < 0.01, Figure 7).

### 3.7. Validation of Candidate Prognostic miRNAs

**Figure 8:**
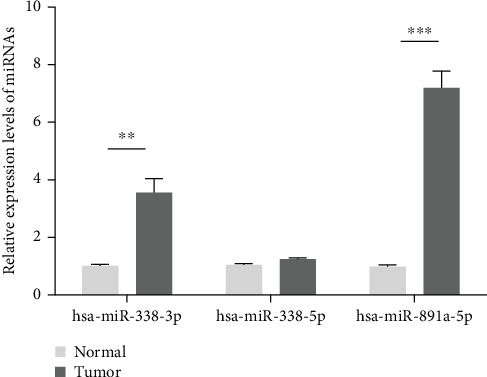
RT-qPCR showing the expression levels of hsa-miR-338-3p, hsa-miR-338-5p, and hsa-miR-891a-5p in DLBCL. ^∗∗^*p* < 0.01; ^∗∗∗^*p* < 0.001.

**Figure 9:**
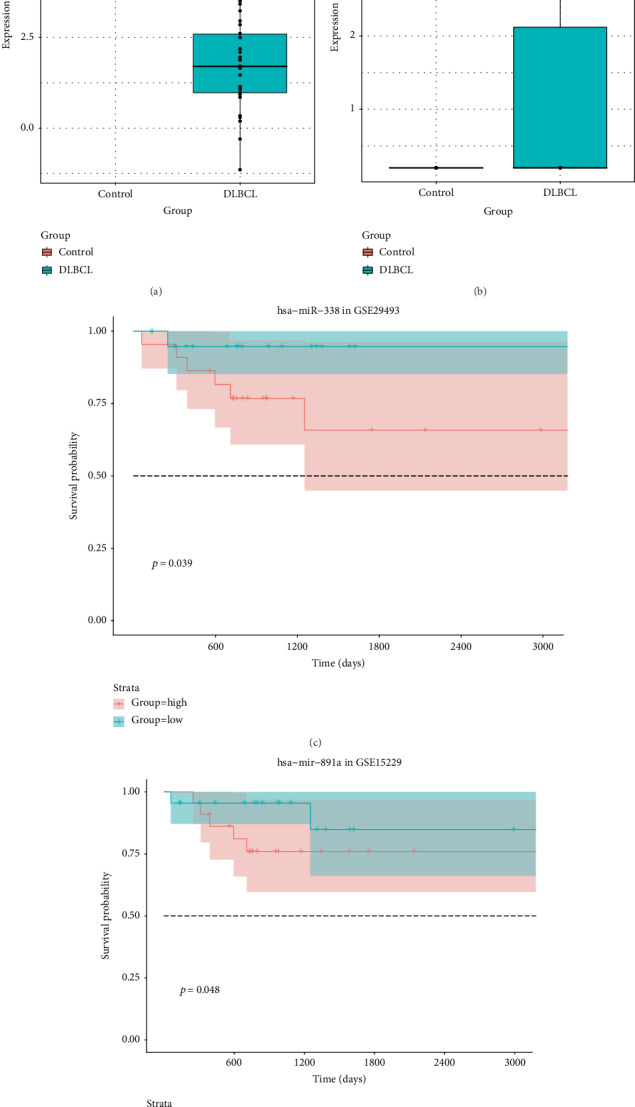
Validation of candidate prognostic miRNAs in independent datasets. The differences in expression patterns of hsa-miR-338 (a) and hsa-miR-891a (b) between DLBCL and controls. Overall survival analysis of hsa-miR-338 (c) and hsa-miR-891a (d) in DLBCL.

Consistent with our bioinformatics results, RT-qPCR results showed that hsa-miR-338-3p (*p* < 0.01) and hsa-miR-891a-5p (*p* < 0.001) were upregulated in DLBCL (Figure 8). Furthermore, we further validated the candidate prognostic miRNAs in independent datasets. Similarly, the results showed that hsa-miR-338 (Figure 9(a)) and hsa-miR-891a (Figure 9(b)) were highly expressed in DLBCL. High expression of hsa-miR-338 (Figure 9(c); *p* = 0.039) and hsa-miR-891a (Figure 9(d); *p* = 0.048) was significantly associated with worse prognosis of DLBCL patients.

## 4. Discussion

As a heterogeneous disease, DLBCL can be classified into activated B-cell, germinal center B-cell, and primary mediastinal B-cell subtypes based on gene expression profiling [21]. Though about 70% of DLBCL patients have survival time longer than five years when treated with immunochemotherapy involving rituximab plus cyclophosphamide, doxorubicin, vincristine, and prednisolone (R-CHOP) [22], however, the remaining patients are still dying of this malignant tumor. In addition, the short- and long-term toxicity of chemotherapy, including secondary malignancies and leukemia, adversely affects the long-term prognosis of patients. Therefore, it is necessary to study new therapeutic targets in DLBCL.

RNA-seq is a next-generation sequencing technology for genome-wide quantitative gene expression, which has some advantages over microarrays in characterizing transcriptomes [23, 24]. However, few studies have investigated the network characteristics of coexpression networks based on RNA-seq. In the present study, RNA-seq and clinical data of DLBCL were retrieved from TCGA. 19 coexpression modules were built by the 12813 most variant genes from 51 DLBCL samples using WGCNA. WGCNA has been proved to be an effective method for detecting coexpression modules and hub genes in many aspects [25]. The interaction between genes in different coexpression modules can be found. Although a large number of biomarkers have been identified and validated, few studies have considered correlations between genes. Genes with similar expression patterns can encode proteins with similar functional properties that can form complexes or can function in similar pathways. Therefore, we made full use of the WGCNA algorithm to detect key genes associated with sample traits in coexpressed gene networks [26]. The purple module was closely related with histology type (*p* value = 1*e*-04). And the cyan module was closely related with age. The incidence of DLBCL is positively correlated with age, about two-thirds of DLBCL patients are over 65 years old worldwide [27]. Research has found that age at diagnosis has a relationship with the molecular features of DLBCL [28].

We found that the genes in the purple module were involved in multiple pathways such as Ras signaling pathway, PI3K/AKT signaling pathway, and cell adhesion molecules. The Ras signaling pathway plays an important role in cancer biology, which regulates cell growth and proliferation. Activating mutations in Ras can result in abnormal activation of its downstream target MEK1/2 [29]. MEK has become a potential target for the treatment of DLBCL. The PI3K/AKT signaling pathway is activated in DLBCL, which plays a key role in controlling the proliferation and survival of DLBCL cells [30]. Activation of this pathway in DLBCL can cause gene mutations, loss of PTEN, or constitutive activation of upstream regulatory pathways [31]. Cell adhesion molecules function in complex biological processes like cancer progression, inflammation, angiogenesis, and metastasis [32–34].

miRNAs are involved in several biological processes by regulating gene expression at the posttranscriptional level, such as cell proliferation and apoptosis [35, 36]. For example, downregulated microRNA-155 promotes cell cycle arrest and apoptosis in DLBCL [37]. And miR-10a suppresses cell proliferation and promotes cell apoptosis via targeting BCL6 in DLBCL [38]. The main reason for the poor results of DLBCL chemotherapy is that DLBCL cells are resistant to chemotherapeutic drugs [39]. It has been confirmed that miRNAs are closely associated with cancer chemosensitivity. To investigate the prognosis value of the genes in the purple module, we made overall survival analysis and results revealed that three miRNAs were closely related with survival. Highly expressed MAFA-AS1 and hsa-mir-338 had shorter survival time than their low expression (*p* value = 0.027 and 0.039, respectively). And low expression of hsa-mir-891a had poorer clinical outcomes than its high expression (*p* value = 0.022). So far, there is no study concerning MAFA-AS1 and hsa-mir-891a. As for hsa-mir-338, it has been reported that hsa-mir-338-3p inhibits invasion and migration of colorectal cancer cells by suppressing smooth expression [40]. Aberrantly expressed hsa-mir-338-3p increases the risk of esophageal cancer [41]. In addition, hsa-mir-338 can be a prognostic biomarker for oral squamous cell carcinoma [42]. Then, we constructed a coexpression network for the three genes by WGCNA. MAFA-AS1 was interacted with 36 genes, hsa-mir-891a was interacted with 11 genes, while no gene showed interaction with hsa-mir-338. Using GEPIA datasets, we found that MAFA-AS1 showed low expression in DLBCL samples (*p* < 0.01).

Taken together, three candidate genes (MAFA-AS1, hsa-mir-338, and hsa-mir-891a) from the purple module could be considered prognostic biomarkers for DLBCL. Compared to IPI, the new prognostic model had some advantages. For example, the three candidate genes could be detected using immunohistochemistry, which is convenient to screen patients with DLBCL and predict their prognosis. Moreover, based on clinical features and candidate genes, it can help improve treatment options, pathobiology, and life expectancy of patients with DLBCL. However, several limitations of our study need to be pointed out. The heterogeneity of the treatment protocols is inevitable, which can bias outcomes. Furthermore, the number of samples is limited; therefore, the prognostic role of the three candidate genes requires to be verified in a larger sample of DLBCL.

## 5. Conclusion

In our study, RNA-seq and clinical data of DLBCL were retrieved from TCGA. 19 coexpression modules were built by WGCNA. And we found that the purple module was closely associated with histological type. Further analysis suggested that three candidate genes (MAFA-AS1, hsa-mir-338, and hsa-mir-891a) were significantly related with clinical outcomes. Therefore, our findings revealed a coexpression module related with histological type and identified three candidate genes (MAFA-AS1, hsa-mir-338, and hsa-mir-891a) that possessed potential value as prognostic biomarkers or potential therapeutic targets of DLBCL.

## Figures and Tables

**Table 1:** Clinical features of DLBCL patients.

Clinical features	Total (*n* = 47)	Alive (*n* = 38)	Dead (*n* = 9)
Age, mean (range)	56.3 (23-82)	56.8 (23-82)	54.1 (27-73)
Gender			
Male	22	17	5
Female	25	21	4
Stage			
I	7	6	1
II	17	15	2
III	5	4	1
IV	12	9	3
-	6	4	2
Race			
Asian	18	14	4
White	28	24	4
Black or African American	1	0	1

**Table 2:** GO enrichment analysis results of genes in the purple module.

Term ID	Ontology	Description	*p*.adjust	Count
GO:0048167	BP	Regulation of synaptic plasticity	0.00573	7
GO:0021782	BP	Glial cell development	0.01269	5
GO:0022010	BP	Central nervous system myelination	0.01269	3
GO:0032291	BP	Axon ensheathment in central nervous system	0.01269	3
GO:0042063	BP	Gliogenesis	0.01776	7
GO:0010975	BP	Regulation of neuron projection development	0.01776	9
GO:0010001	BP	Glial cell differentiation	0.01902	6
GO:0085029	BP	Extracellular matrix assembly	0.02101	3
GO:0048708	BP	Astrocyte differentiation	0.02214	4
GO:0050804	BP	Modulation of chemical synaptic transmission	0.02299	8
GO:0099177	BP	Regulation of transsynaptic signaling	0.02299	8
GO:0060291	BP	Long-term synaptic potentiation	0.02542	4
GO:0014002	BP	Astrocyte development	0.02736	3
GO:0110110	BP	Positive regulation of animal organ morphogenesis	0.02776	4
GO:0001505	BP	Regulation of neurotransmitter levels	0.03131	7
GO:0014003	BP	Oligodendrocyte development	0.03886	3
GO:0001657	BP	Ureteric bud development	0.03886	4
GO:0072163	BP	Mesonephric epithelium development	0.03886	4
GO:0072164	BP	Mesonephric tubule development	0.03886	4
GO:0001504	BP	Neurotransmitter uptake	0.04008	3
GO:0001823	BP	Mesonephros development	0.04097	4
GO:0050768	BP	Negative regulation of neurogenesis	0.04282	6
GO:0006953	BP	Acute-phase response	0.04605	3
GO:0016264	BP	Gap junction assembly	0.04605	2
GO:0071281	BP	Cellular response to iron ion	0.04605	2
GO:0014013	BP	Regulation of gliogenesis	0.04823	4
GO:0051961	BP	Negative regulation of nervous system development	0.04823	6
GO:0042552	BP	Myelination	0.04823	4
GO:0007272	BP	Ensheathment of neurons	0.04823	4
GO:0008366	BP	Axon ensheathment	0.04823	4
GO:0043209	CC	Myelin sheath	0.00032	7
GO:0014069	CC	Postsynaptic density	0.00777	7
GO:0032279	CC	Asymmetric synapse	0.00777	7
GO:0099572	CC	Postsynaptic specialization	0.00777	7
GO:0098984	CC	Neuron to neuron synapse	0.00777	7
GO:0097449	CC	Astrocyte projection	0.02459	2
GO:0043083	CC	Synaptic cleft	0.02892	2
GO:0098793	CC	Presynapse	0.03602	7
GO:0031012	CC	Extracellular matrix	0.03602	7
GO:0033267	CC	Axon part	0.03871	6
GO:0030426	CC	Growth cone	0.04208	4
GO:0030427	CC	Site of polarized growth	0.04208	4
GO:0097386	CC	Glial cell projection	0.04208	2
GO:0150034	CC	Distal axon	0.04208	5
GO:0034364	CC	High-density lipoprotein particle	0.04689	2
GO:0048306	MF	Calcium-dependent protein binding	0.01134	4
GO:0005539	MF	Glycosaminoglycan binding	0.01134	6
GO:0048018	MF	Receptor ligand activity	0.01134	8
GO:0008201	MF	Heparin binding	0.01134	5
GO:0004857	MF	Enzyme inhibitor activity	0.01134	7
GO:0005381	MF	Iron ion transmembrane transporter activity	0.02116	2
GO:1901681	MF	Sulfur compound binding	0.04249	5

BP: biological processes; CC: cellular components; MF: molecular functions.

**Table 3:** KEGG pathway enrichment analysis results of genes in the purple module.

ID	Description	*p*.adjust	Count
hsa04721	Synaptic vesicle cycle	0.01171	2
hsa04014	Ras signaling pathway	0.01222	3
hsa04727	GABAergic synapse	0.01507	2
hsa05032	Morphine addiction	0.01572	2
hsa04514	Cell adhesion molecules (CAMs)	0.03700	2
hsa04151	PI3K-Akt signaling pathway	0.03718	3
hsa04723	Retrograde endocannabinoid signaling	0.03890	2
hsa05226	Gastric cancer	0.03938	2
hsa00340	Histidine metabolism	0.04811	1
hsa04614	Renin-angiotensin system	0.04811	1

**Table 4:** Three candidate genes related with prognosis of DLBCL.

Gene	Hazard ratio	95% CI	*p* value
MAFA-AS1	3.794	0.96-14.995	0.026604
hsa-mir-338	4.185	1.127-15.54	0.039354
hsa-mir-891a	0.205	0.055-0.772	0.021503

## Data Availability

The datasets analyzed during the current study are available from the corresponding author on reasonable request.

## Abbreviations

DLBCL: Diffuse large B-cell lymphoma
IPI: Prognostic index
RNA-seq: RNA sequencing
TCGA: The Cancer Genome Atlas
WGCNA: Weighted gene coexpression network analysis
FPKM: Fragments per kilobase of transcript per million mapped reads
MAD: Median absolute deviation
ME: Module eigengene
GO: Gene Ontology
KEGG: Kyoto Encyclopedia of Genes and Genomes
GEPIA: Gene Expression Profiling Interactive Analysis
BP: Biological processes
CC: Cellular components
MF: Molecular functions.
